# Victimization of Applicants for International Protection Residing in Belgium: Sexual Violence and Help-Seeking Behavior

**DOI:** 10.3390/ijerph191912889

**Published:** 2022-10-08

**Authors:** Lotte De Schrijver, Anne Nobels, Jonathan Harb, Laurent Nisen, Kristien Roelens, Tom Vander Beken, Christophe Vandeviver, Ines Keygnaert

**Affiliations:** 1 International Centre for Reproductive Health, Department of Public Health and Primary Care, Ghent University, 9000 Ghent, Belgium; 2 Department of Psychiatry, Ghent University Hospital, 9000 Ghent, Belgium; 3 CARE-ESPRIst, Études et Évaluations, University of Liège, 4000 Liège, Belgium; 4 Department of Obstetrics and Gynecology, Ghent University Hospital, 9000 Ghent, Belgium; 5 Department of Human Structure and Repair, Ghent University, 9000 Ghent, Belgium; 6 Department of Criminology, Criminal Law and Social Law, Institute for International Research on Criminal Policy, Ghent University, 9000 Ghent, Belgium; 7 Research Foundation—Flanders (FWO), 1000 Brussels, Belgium

**Keywords:** sexual and gender-based violence, migrants, refugees, asylum seekers, Europe, prevalence, Belgium

## Abstract

Background: Sexual violence (SV) literature on applicants for international protection (AIPs) shows that they are at high risk of victimization. The study objectives are to provide an exploratory overview of the occurrence of SV in AIPs in Belgium and their help-seeking behavior (HSB). This overview is crucial to develop prevention strategies and care paths focusing on providing adequate care to AIP SV victims in Belgium. Methods: Quantitative data from structured interviews with AIPs (*n* = 62) triangulated with qualitative data from in-depth interviews with AIP SV victims (*n* = 11) served to explore the nature and impact of SV in AIPs in Belgium and their HSB. Results: A total of 83.9% of respondents have experienced SV. A total of 61.3% were victimized within the past year. Victimization seems more gender-balanced than in the general population. AIPs link SV to their legal status and their associated vulnerable situation. HSB upon SV was very limited in this sample. Help-seeking barriers interfering with the decision-making process to consult (in)formal resources were identified. Conclusions: AIPs in Belgium are at high risk of SV. Despite the impact of SV on AIPs’ lives, HSB upon SV is rare. The provision of age-appropriate sexual education and development of policies that will reduce help-seeking barriers is needed.

## 1. Introduction

Sexual violence (SV), defined by the World Health Organization (WHO) as “every sexual act directed against a person’s will, by any person regardless of their relationship to the victim, in any setting” [1], is a widespread problem threatening public health [2]. It consists of different types of violence, including sexual harassment without physical contact, sexual abuse with physical contact but without penetration, and (attempted) rape [3]. Sexual victimization has a profound impact on victims’ physical, mental, sexual, and reproductive health [2,4,5]. The international sexual violence literature on applicants for international protection (AIPs) suggests that they may be more frequently exposed to sexual victimization than the general population [6,7,8,9]. Worldwide sexual violence prevalence numbers in AIPs are to date unavailable. Reported estimates range up to 99.8% [9] and strongly depend on the included target population, the study design and adopted definition of sexual violence [6,9]. Building further along this line, we can state that robust European prevalence figures are also lacking [6].

In the limited available studies on sexual victimization in AIPs in Europe, AIPs report sexual violence exposure in their country of origin, while en route, and upon arrival in host countries. In a study from Doctors of the World (Médecins du Monde, MdM) in 10 European countries and Canada, 21.1% of the reported rapes and 17.7% of the reported sexual assaults took place after the arrival of the victim in the host country [10]. In another study that looked into sexual and gender-based violence in eight European countries (i.e., Belgium, Greece, Hungary, Ireland, Malta, the Netherlands, Portugal, and Spain), researchers observed direct sexual and gender-based violence exposure in 58.3% and indirect exposure in 76.6% of the participating refugees, AIPs, and undocumented migrants [11]. A study on sexual and gender-based violence in refugees, AIPs, and undocumented migrants in Belgium and the Netherlands, found that 56.6% of the participants were exposed to sexual violence [12]. In one in five cases, the exposed participant had been sexually victimized [12]. A more regional study of AIPs residing on Lesvos in Greece, showed that 35% of the reported cases of sexual victimization took place in the country of origin; almost half of the cases occurred during transit, and 5% of the sexual violence cases happened upon arrival on Lesvos [13]. These figures hint at AIPs being at risk for sexual victimization, yet they cannot be considered as robust and representative prevalence numbers because these study designs do not allow for extrapolation to the total group of AIPs in the relevant regions [6].

Most studies have focused on female victims alone [9], yet some studies indicate that AIPs’ sexual victimization and perpetration may be more gender-balanced in comparison with the general population, with men and women reporting a comparable prevalence of sexual violence [11]. Furthermore, European citizens appear to be an important number of the assailants of sexual violence against AIPs [9,11,12,14]. Often, the assailants were known to the victims [9,11,12,13] or held a position of power over them [9,11,12].

The impact of sexual victimization on their lives may also be far greater than in the general population because of specific risk factors emerging from the vulnerable situation AIPs find themselves in [15]. Identified vulnerabilities for sexual victimization include having a low socioeconomic status, being previously exposed to violence, having a poorer (mental) health status, lacking support and protection from social and familial networks, and residing in reception and accommodation facilities, in social housing, or being homeless [7,15,16,17,18]. AIPs may not only experience interaction effects of different vulnerabilities, but they also perceive more barriers for seeking help upon sexual victimization [15,19,20].

Many European countries, have been under higher migratory pressure in the past several years. To develop prevention strategies targeting sexual victimization in AIPs and care paths focusing on providing adequate care to applicant for international protection (AIP) victims of sexual violence, having an overview of the occurrence of sexual victimization and help-seeking behavior (HSB) upon sexual victimization in this population is crucial. Aside from knowing the magnitude of the problem, understanding the impact of the phenomenon and the associated HSB is vital. Furthermore, insights into the decision-making process underlying seeking help upon sexual victimization is essential to provide care matching the victims’ needs [21]. In order to contribute to covering these knowledge gaps, we conducted an exploratory mixed-methods study in Belgium. Based on quantitative data collected from a randomly selected sample of AIPs, we aimed to get an indication of the nature of sexual violence in this population. Moreover, to contextualize the quantitative findings and to understand the underlying mechanisms, we triangulated these insights with findings from a qualitative study by using semi-structured in-depth interviews for complementarity.

The objective of this study was to answer the following research questions:

1. Are AIPs residing in Belgium vulnerable to sexual victimization?
2. In which context does sexual victimization in AIPs occur?
3. How does sexual victimization impact AIPs?
4. Do AIPs residing in Belgium seek help upon sexual victimization?

## 2. Methods

### 2.1. Ethical Considerations

This study was approved by the Commission for Medical Ethics of Ghent University Hospital/Ghent University (B670201837542). It was designed and performed in line with the principles of the Declaration of Helsinki. This study only included participants of 16 years and older given ethical and practical regulations related to the legal age of consenting to sex, which is 16 years old in Belgium. All participants gave informed consent before initiating the online survey. Additional informed consent was given before starting the in-depth interviews.

### 2.2. Research Design

This study was conducted by using a mixed-methods explanatory sequential design. This design implies that quantitative data is first collected followed by qualitative data in two consecutive phases of one study [22,23]. Such a design allowed us to take advantage of the strengths of each individual method employed while mitigating their weaknesses [24,25]. This explorative mixed-methods study also allowed for more holistic inferences and an opportunity for triangulation for complementarity. This type of triangulation starts from the perspective that the positionality of the observer is of importance in the analysis of the results and that datasets may not necessarily produce the same understanding of the object of observation [26]. As such, by combining data collected via different methods and/or via different observers, complementary triangulation is used to provide a more complete image of the research question [26]. Furthermore, this design also allows to illustrate and provide context through mutually corroborated findings though different sets of data [27].

The first phase of the mixed-methods study entailed a quantitative study using a survey. This survey was conducted via laptop- or tablet-assisted face-to-face structured interviews. In preparation of the quantitative and qualitative study, we conducted a critical interpretive synthesis (CIS) of relevant literature (see [6,15]). The findings from the CIS gave input to this phase by highlighting the research challenges that had to be considered to reach migrant populations and to make it possible to compare the findings with quantitative studies on sexual violence in the general population (see e.g., ref. [28]). In addition, the CIS helped identify crucial content for the survey on the mechanisms, nature, magnitude, and impact of sexual victimization in AIPs. The quantitative phase was essential to provide numeric data on the occurrence of sexual victimization in AIPs residing in Belgium and to identify correlates of sexual victimization. To complement, contextualize, and deepen the findings from the quantitative survey, a subsequent qualitative study followed as a second phase of this mixed-methods design. The qualitative phase entailed semi-structured in-depth interviews with AIP victims. Findings from the qualitative analysis were used to delve more deeply into the mechanisms underlying sexual violence and the help-seeking behavior upon sexual victimization.

### 2.3. Measures

#### 2.3.1. Quantitative Data Collection

The initial goal of the quantitative study was to conduct a self-report survey probing sexual victimization and perpetration among a randomly selected sample of AIPs aged 16 to 100 residing in any of the asylum reception facilities in Belgium, regardless of gender or sexual orientation. AIPs are a hard-to-reach population. Therefore, data were collected via face-to-face interviews by trained interviewers to increase the response rate and to allow persons with lower literacy levels to participate [29]. The survey was available in Arabic, Dari/Farsi, Dutch, English, French, and Pashtu. These languages were selected based on the most common languages that were spoken in Belgium (i.e., Dutch, English, and French) and the most commonly spoken languages, aside from Dutch, English, and French, among registered AIPs in Belgium at the moment of recruitment (i.e., Arabic, Farsi/Dari, and Pashtu).

In the preparatory CIS regarding the design and comparison of sexual victimization prevalence studies in AIPS, one of the main problems identified was the lack of transparency about the definition of sexual violence used in the studies and potential associated interpretation bias. In this study, sexual violence was defined according to the WHO definition (cf. supra) [1]. As is recommended internationally, behaviorally specific questions [30,31,32,33] were used to provide reliable estimates of both female and male sexual victimization [33,34] within different cultures.

The questionnaire consisted of 13 modules and was designed to maximize sexual violence (i.e., victimization and perpetration) disclosure by starting with less sensitive topics, building up toward the sexual violence modules. Both lifetime SV experiences and SV experienced in the past 12 months were assessed. The behaviorally specific questions were derived from the revised Sexual Experience Survey (SES-R) [31,35], the Sexual Aggression and Victimization Scale (SAV-S) [36], and the Senperforto questionnaire [12]. All questions on sexual violence were adapted to the Belgian social and legal context. The process of developing this survey has been described elsewhere (see [33,37]).

To explore the impact and help-seeking behavior upon sexual victimization, potential associated factors were measured. To measure mental health outcomes, the following validated scales were used: the Patient Health Questionnaire (PHQ)-9 for depression [38] (Cronbach alpha (α) = 0.829), the General Anxiety Disorder (GAD)-7 for anxiety (α = 0.857) [39], the Primary Care PTSD Screen for DSM-5 (PC-PTSD-5) for PTSD [40], and the Alcohol Use Disorder Identification Test-Short version (AUDIT-C) to assess hazardous alcohol use [41]. We integrated these scales as, in their validated form and with accompanying norm scales, they were thus far the best suited to both the general population and the AIPs. In addition, participants were asked about sedative use, cannabis use, illegal drug use, and self-harm and suicide attempts, both during their lifetime and in the past 12 months.

Furthermore, participants were asked questions regarding potential barriers, satisfaction, and their experiences with (in)formal disclosure and help-seeking from professional services or the police as it related to the sexual violence incident that they identified as having had the most impact on their lives. If they experienced a single incident, this was automatically identified as the incident of reference.

#### 2.3.2. Qualitative Data Collection

Participants were recruited via the survey conducted during the first quantitative data collection phase (*n* = 62). Survey participants who indicated to be (in)directly exposed to sexual violence were asked at the end of the survey whether they would agree to be invited to participate in an in-depth interview regarding their experiences with sexual violence. Consenting participants were informed about the nature, the duration, and procedure of the study, and informed consent was obtained. Participants were assured that they could stop the interview at any time, that the study would not hold risks or costs for them, and that they would be insured during the interview. Furthermore, they were informed about the steps taken to protect the confidentiality of all the information they would provide. Participants were not offered compensation for their participation, but received a small gift (i.e., cookies or hand soap) after having completed the interview.

Data were collected through face-to-face semi-structured in-depth interviews (*n* = 11). The first nine interviews were carried out by a female researcher with a background in clinical psychology and sexology and with the help of a male Arabic-speaking interpreter. These interviews took place between January 2020 and 12 March 2020. Two additional interviews were conducted in January 2021 by an Arabic-speaking male clinical psychologist. At that time, the ruling social distancing measures related to the COVID-19 pandemic did not allow for face-to-face interviews to take place. Therefore, these two interviews took place by telephone. However, another real face-to-face interview took place in the same period, but under different circumstances. In these particular circumstances, the social distancing measures at that time did allow a face-to-face interview. This interview was conducted by a female interviewer with a background in medical anthropology and public health with extensive experience in conducting interviews on sexual violence in this population. During the analysis, we verified whether the face-to-face and phone interviews were of the same quality. During a blind check, we could not distinguish the face-to-face interviews from the interviews conducted over the phone. We thus found that they were of the same quality.

The semi-structured in-depth interviews were guided by a topic list containing seven themes: (1) framing sexual violence, (2) (in)direct exposure to sexual violence, (3) perceived reasons for victimization and perpetration, (4) consequences of sexual victimization, (5) coping and help-seeking behavior, (6) impact on family and peers/transgenerational transmission, and (7) recommendations for prevention and care upon sexual victimization. To guarantee anonymity, sociodemographic information was collected before the start of the interview and the audio recording. To start the interviews in a standardized manner, each interview started with a question to define what sexuality means for the interviewee, followed by the same question regarding “sexual violence” (theme: framing sexual violence).

Interviews lasted between 53 min and 1 h 51 min. The interviews were conducted in Arabic, English, and French. Citations reported in this paper were translated into English.

### 2.4. Sample

In the first and quantitative phase of this mixed-methods study, the sample of AIPs was drawn from the databases of the collective and individual reception structures responsible for organizing the reception facilities for AIPS residing anywhere in Belgium. The head offices of the reception structures (Fedasil, Rode Kruis, and Croix-Rouge) drew a random sample, based on the criteria provided by the research team, such as legal status, age, gender, and nationality. The survey was available in seven languages (English, French, Dutch, Arabic, Dari/Farsi, and Pashtu). Because data regarding language was not included in the databases, nationality was used as a proxy to sample respondents who were likely to speak at least one of these languages. As a result, 142 nationalities were included based on a list of countries where at least one of the languages was spoken. The description of the sampling procedure and recruitment strategy and the lessons learned from developing and applying this research design have been published elsewhere (see [28]).

As a result of the COVID-19 pandemic and the enforced lockdown measures to prevent its spread, we were forced to stop the data collection on the 12 March 2020. By this date, 62 structured interviews with AIPs were concluded. As such, quantitative data was collected in 62 AIPs, whose average residence time in Belgium was 10.8 months. Three out of four participants were assigned the male sex at birth. The sociodemographic information regarding our study population can be found in Table 1. No significant differences in sociodemographic characteristics were found when comparing SV victims to those participants who did not experience SV (*p* > 0.1).

In the qualitative phase of the study, a total of 12 AIPs participated in the in-depth interviews. However, one interview was interrupted and had to be postponed. In the end, the participant dropped out during the second part of the interview. Hence, 11 interviews were included in the analysis. The sample consisted of four heterosexual women and seven heterosexual men between the age of 23 and 53 years old who were (in)directly exposed to sexual violence. They all resided in asylum reception centers located in Flanders, the Northern part of Belgium.

### 2.5. Data Analysis

#### 2.5.1. Quantitative Data Analysis

Statistical analysis was performed by using SPSS Statistics 26. Descriptive statistics were used to illustrate the sociodemographic characteristics, sexual victimization, mental health status, and help-seeking behavior upon sexual victimization of the study sample. Sexual violence variables were grouped into hands-off (eight items) and hands-on (nine items) sexual violence, the latter being further grouped into sexual abuse (four items) and attempted or completed rape (five items). We created dichotomous variables out of all items to assess lifetime SV victimization and victimization in the last 12 months. A detailed overview of the sexual violence outcome measures can be found in the Supplementary Materials.

The variables measuring barriers to contact professionals were grouped into reasons linked to the victim (five items), other persons (three items), accessibility (two items), and other reasons (one item). The variables measuring barriers to contact the police were grouped into reasons related to the victim (four items), the assailant (one item), accessibility (one item), the police (five items), and other reasons (one item). We created dichotomous variables out of all items to assess help-seeking behavior upon sexual victimization.

We conducted a bivariate analysis to compare the proportion of sexual victimization within the sociodemographic categories, the different categories of depression, anxiety, resilience, PTSD, alcohol and drug use, suicide attempts and self-harm, as well as to compare sexual victimization rates in the male and female subgroups using a Chi-square test or a Fisher exact test if the assumptions of the Chi-square test were not met. The *p*-values are presented. To compare the means of the continuous variables, the independent samples *t*-test was used. All assumptions were checked. Levene’s test was used to check for homogeneity of variance, which led to the use of the Welch *t*-test statistic, as equal variances could not be assumed.

#### 2.5.2. Qualitative Data Analysis

The semi-structured in-depth interviews (*n* = 11) were transcribed ad verbatim. The accuracy of the transcriptions was verified by a researcher other than the one who made the transcription by comparing them to the audio recordings before the start of the first coding round. Initial ideas for analysis were noted down. The verified text files were processed and analyzed by using the software NVivo 12.

A thematic analysis inspired by Braun and Clarke’s protocol [42] was carried out without a predetermined coding scheme. A first theoretical code tree was developed based on themes central to the topic list guiding the in-depth interviews (which was based on the CIS (see [15])) supplemented with identified recurring themes that came forward after a few interviews. Starting from this baseline code tree, the researchers continued with an inductive coding strategy which involved a line-by-line coding with codes derived from the narratives. All interviews were coded by at least two researchers individually. The code trees from the individual researchers were merged into one. The merged code tree further served as the backbone of the analysis. The codes were collated into potential themes, which were in turn reviewed to ultimately result in a thematic map for analysis [42]. In different loops of analysis, the themes were refined and regrouped according to key ideas until the overall story of the analysis emerged and became clear and definable.

## 3. Results

Both quantitative and qualitative methods were used to address each of the four research questions. Below we present the quantitative findings followed by the qualitative findings when both are available to answer a certain research question.

### 3.1. Sexual Victimization in Applicants for International Protection Residing in Belgium

#### 3.1.1. Nature of Sexual Victimization

Table 2 gives us an overview of the occurrence of both hands-off and hands-on sexual victimization in our quantitative sample (*n* = 62). Most AIPs have experienced sexual victimization in their life (83.9%) as well as in the past 12 months (61.3%). Most participants who reported sexual victimization in the past 12 months, and most likely had at least one victimization experience in Belgium, because the average time our study participants were in Belgium was 10.8 months. Table 3 shows the distribution for any sexual victimization, hands-off sexual victimization, hands-on sexual victimization, sexual abuse, and rape for the lifetime and the past 12 months per sex at birth. The same trends in occurrence are found in both male and female victims.

#### 3.1.2. What Is Sexual Violence According to Applicants for International Protection?

When measuring sexual violence, it is important to define the measured concept. In the quantitative survey, we used behaviorally specific questions to objectively measure sexual violence exposure. The behaviorally specific questions are mostly based on research from the U.S. and Europe. As such, interviewees were asked in the qualitative study to explain to us what they considered to be sexual violence. It appears that AIPs from different cultures, religions, and nationalities define sexual violence in comparable ways. Four returning defining elements were identified.

*Without consent.*

Overall, sexual violence was defined as sexual behavior that occurred without the consent of at least one of the persons involved. As Cristiano (25 years old) said: “It is actually having a sexual contact with someone against his will or against her will.” The interviewed AIPs identified that it could occur in any type of relationship, including marriages, and against both men and women. Rahi (42 years old) for example explained that “although he is her husband”, when a man does not respect his wife and just “takes what he needs” without considering her, this also accounts as sexual violence. According to the participants, it is not the context in which the sexual behavior takes place, but rather the experience of the persons engaged in it, that provides the core elements to determine whether or not it concerns sexual violence.

*“Like animals”.*

Several AIPs made the comparison to the lust-driven manner in which animals have sex. Elen (48 years old) pointed out that “you can actually compare it to animals that actually interact with each other; without any…without any emotion. You can compare it with yes, animals, animal attitude in principle.… People who are solely looking for action and then be done. As I said before, who treat each other like animals.” The description “like animals” was literally used by several AIPs in different languages to explain sexual violence. They further described it as a way of having sex just for the act of sex itself, not out of love or with respect for one another.

*Without equality & voluntariness.*

Respect and equal power relations between sexual partners was often mentioned as a necessary condition for a healthy sexual experience. Interviewees defined sexual violence as (1) sexual behavior without respect for one another, as (2) sexual relations in which there are “unequal” power positions, and as (3) sexual behavior that takes place by means of coercion.

“Sexual violence to me is, any sexual act aims, like one side wants it but the other side refuses it and it happens whether due to pressure on them, or by hitting them, so the other side is not, like one side is accepting and the other is not.”

(Ahmad, 31 years old)

*Multiple forms.*

AIPs agreed that sexual violence could be both reflected by hands-off (e.g., Elen (48 years old, who stated that “…the speaking can already be a form of sexual violence in principle. It doesn’t necessarily have to be touching or it…the act in itself, so there are other forms of sexual violence”) and hands-on behavior (e.g., Majohoul, 33 years old, who stated that For me personally that is actually a kind of rape, because it is not only the act itself, but also kisses or touch or the act itself. That’s all the same, that comes down to the same thing. Ultimately, that’s kind of rape”). They consider it to encompass more than being penetrated against one’s will. According to them, sexual violence also includes unwanted sexual comments, showing unwanted sexual content, and unwanted sexual touching, and this can be online and/or in real life.

#### 3.1.3. Who Are the Assailants of Sexual Violence against AIPs?

Table 4 presents the gender distribution of assailants related to lifetime sexual victimization or sexual victimization in the past 12 months. Table 5 shows the relationship of the victim to the assailants. These tables demonstrate that AIPs identified their assailants as being women as much as men. Moreover, around 60% of the sexual victimization incidents are perpetrated by someone unknown to the victim.

### 3.2. Context of Sexual Violence

AIPs reported sexual victimization in the country of origin, during transit, and after arrival in Europe and Belgium. Some participants testified in the in-depth interviews about direct and indirect exposure to sexual violence during their childhood. In those cases, it mainly concerned domestic sexual violence or child abuse perpetrated by or toward someone of their community. One interviewee was sexually victimized in relation to conflict in the country of origin. Sexual victimization during migration concerned incidents mostly in refugee camps within the migrant group and sexual exploitation by human traffickers or by locals who “offered to help them.” Once in Europe and/or Belgium, AIPs seemed to be mostly, but not exclusively, victimized by locals. Cristiano (25 years old) for example, experienced sexual victimization in Brussels by locals and in Italy by an AIP with whom he shared a room in the reception center where he was staying. He explained: “Actually, I have often experienced it personally. I don’t actually talk about it a lot, but I did experience several things personally in the Maximilian Park, so in Brussels…. I actually experienced that in Italy, when someone wanted to assault me with violence or have a sexual relationship…. They know…they sense that, for some reason, they know that we are actually refugees here…. The majority of people who are staying in the park have the same problem. Also experienced such things.” From Cristiano, but also from other interviewees, we learned that most of them had experienced at least one incident in Belgium. Sexual violence against AIPs appeared almost like such a common practice that they tend to normalize its occurrence. Youssef (31 years old) described that after being victimized while he was living in Brussels under a bridge at Gare du Nord, he started to consider the threat of sexual violence as part of his life while being an AIP: “Because that’s everyday there, we’re used to that. So, I discussed it with my friends, what had happened to me. And those friends also said, ‘Yes that is [common] here, you know that is very normal here.’”

Grooming AIPs by locals with the promise of, among other things, offering shelter or a shower/bath seemed to be a common practice in the Brussels area where AIPs need to register upon arrival in Belgium. Interviewees explained how, as AIPs, they are vulnerable to sexual victimization (e.g., Cristiano’s and Youssef’s testimonies, supra and infra) based on their own experiences and those of other AIPs they know. Throughout the interviews, AIPs described how they or people they know (e.g., family, friends, peers) were exploited because of their difficult socioeconomic situation. Many respondents highlighted that those refugees who are homeless or undocumented are very vulnerable to different types of violence.

“…but you know, we are actually very, we are fragile there, we are very vulnerable in those places…. I think the homeless, they suffer even more than refugees. Refugees have some kind of shelter anyway. They can go somewhere for, like shelter or they get food or…. But homeless people, I think homeless people go through much, um, much more than we do, as refugees…. Indeed, as long as you don’t have valid papers or provisional papers or…. then danger lurks around every corner.”

(Youssef, 31 years old)

“They know our weaknesses, they think we are in a weak position, and they actually want to take advantage of that…. Yes, they know of course that we live on the streets. That we have to spend the night somewhere. They sometimes offer alternatives, or even money, also money to go with them. But yes, that is actually a bit painful, because that is not what we are here for either. I have other concerns on my mind. I am a refugee.”

(Cristiano, 25 years old)

Aside from their legal status, being homeless was another factor that AIPs considered to make them increasingly vulnerable to sexual violence. On the other hand, some AIPs also indicated that staying in a reception center put them at higher risk. First, this is because it gives outsiders the chance to identify the AIPs as AIPs. Elen (48 years old) for example, described how one day a neighbor of the center she was staying in offered her a ride back from the store to the reception center. While she was in the car, the man propositioned her to have sex in exchange for a couple of euros. When she refused, he became violent. She added: “most think ‘uh those people are here in the center just for the sake of money’ or… they think ‘those people, uh, they are not looking for safety. Uh, they’re just here to make money and profit and so they’re willing to do anything’. So that’s a very wrong impression.”

Secondly, reception centers were considered places of high risk for victimization because sometimes it’s impossible to protect oneself in a reception center. AIPs residing in “open centers” described feeling unsafe because anyone can enter without them being identifiable as visitors. Maryam (53 years old) initially felt safe when living in a reception center, but soon this changed: “Normally, because only families stay here, it is normally safe, in parentheses. So, we don’t always lock the door, we don’t lock the door. But that was then five o’clock in the morning. And someone then sneaked in and lay down on the girl and started doing all kinds of actions.”

Majohoul (33 years old) said that as an AIP in a reception facility, one should always be on guard. He added: “Yes, it is actually pure coincidence, because, if you live in refugee camps or centers, that is, you are just, yes, you are just a prey basically. Anything can happen to you there, anyone can experience those situations.”

### 3.3. Impact of Sexual Victimization on Applicants for International Protection

Sexual victimization has an impact on the lives of AIP victims of sexual violence, though the experienced impact of their exposure to sexual violence differs from one victim to the other. Overall, sexual victimization impacts the AIP victims’ physical, psychological, sexual, reproductive, and socioeconomic realities. Fatoumata (23 years old) shared the following: “I’m not going to lie. I have trouble sleeping. I don’t feel well at all. I’ve passed some [things] that won’t leave me…. I see it and when I talk about it, it reminds me of all the details”. Psychological and sleeping problems were regularly mentioned in the in-depth interviews, as were difficulties in trusting others which impaired social relationships with their family, their peers but also with professionals in reception centers. Questioning one’s life and experiences, feelings of frustration, hatred, and revenge were common among AIPs victims. Also, questions on their own gender identity, gender roles, and sexual orientation were present. Maryam (53 years old) said “I didn’t consider myself as a woman” when she explained how sexual violence had impacted her. Youssef (31 years old) also reported questioning how others perceive his sexual and gender identity: “Yes, I have asked myself the question, ‘Is it maybe because, those people [men who are attracted to men] are attracted to me or because they find me beautiful or something?’ I’ve asked that question, too.”

Reported long-term consequences are worrying and ruminating post-sexual victimization. Seeking answers to the question “why did this happen to me” and mentally exploring ways to avoid further violence and ways to cope with the consequences often appear central to the worrying and rumination. The following quote from Ali (26 years old), a victim of conflict-related sexual violence, is illustrative here:

“Why he [assailant] broke me, why I am here? Why in Turkey what happened to me, in Greece what happened to me?… Why I lost my money on my way, why the smugglers take my money and they put me in the boat. I stay one day in the water, I become in one island for one year and nine months, in like prison. I sleep in the tent. Why all this happening to me?… Why I become refugee?… But what I should be: next to the people they need me. It’s something like happy idea and I am here, and I left. I left my life, my stories, my everything, my mom, my father.”

(Ali, 26 years old)

In terms of impact, the consequences and the stigma around sexual victimization could lead to total social rejection by one’s community or family. Interviewees also explained how both victims and assailants may be killed in some cultures to cope with sexual victimization. Most AIP victims talked about stigma and shame related to the sexual victimization. In some cases, exposure to sexual victimization has forced them to become a refugee or to hide certain parts of their identity.

Efforts to conceal the violence and to avoid the stigma associated with it, had far-reaching and long-term consequences for AIP victims, not only psychologically, but also socioeconomically and in terms of experiencing difficulties building social relations, barriers for healthy sexual relationships, exposure to forced pregnancies and marriage, and difficulties in taking up parental roles.

For example, one interviewee explained how she fell pregnant from an older community member who had raped her when she was 15 years old. To avoid bringing shame to the family, her parents forced her to marry her rapist and keep the child. Therefore, this led to a disruption of the relationship with her parents and the development of serious trust and attachment issues. These issues and the lack of a trusted maternal role model ultimately compromised the mother–daughter relationships she developed with her own daughters and her capacity to function as mother/caregiver to the daughter who she regarded as an outcome of her rape. Fatoumata (23 years old) explained: “At the beginning, when it happened, I saw everything. It was horrible. I could not take care of my children, because my daughters, I had by his rapes.” Furthermore, she expressed her difficulties with her self-image as a woman, a mother, and her struggles with depressive symptoms and feelings of powerlessness/helplessness.

Most of the interviewees indicated worrying about avoiding sexual victimization and other forms of violence, in addition to their worries about their migratory process. Youssef (31 years old) expressed his frustration about the circle of violence he experienced associated with migration. He felt that fleeing from violence only made him (and others) more vulnerable to further violence. He expressed the following:

“…We actually fled our country because of violence, war, unsafety, a lot of negative things and we are here in Europe to, to find a safe haven actually. Peace! We don’t want violence, we don’t want to be raped, we don’t want to be assaulted. So those are things that we don’t want at all. So, our goal is just to have a normal and safe life in Europe.”

(Youssef, 31 years old)

Table 6 presents the quantitative findings regarding mental health of AIPs. Overall, participants reported poor mental health. However, different from what was expected based on the results of the qualitative study, we found no difference in the proportion of sexual victimization between the categories of the different mental health outcomes. This indicates that being sexually victimized during one’s lifetime does not impact the current mental health status of AIPs in Belgium.

To cope with sexual victimization, AIPs reported having strategies in place to protect themselves from all kinds of violence. By hiding parts of their identity and behaving differently in public, they try to avoid attracting unwanted attention. Elen (48 years old) gave an example of how she concealed her identity to protect herself. She said: “I actually have two, yes two personalities actually basically, so then….to the outside world I behave differently than when I am alone in my room or something, then I am my own.” Furthermore, some AIP victims described hypervigilance because of their victimization experiences. They try to be in control by means of making contingency plans. This includes Ali (26 years old), for example:

“I am not stupid also, I’m trying to be smart, I’m thinking, I have a plan, always when I see the door for example. I do plan, and I see close the border, the border is closed, I try to pass the way, to find the border or find the place I can move, or I can do my plans.”

(Ali, 26 years old)

### 3.4. Help-Seeking Behaviour of Applicants for International Protection Residing in Belgium upon Sexual Victimization

#### 3.4.1. Do Applicants for International Protection Residing in Belgium Disclose Sexual Victimization?

Table 7 presents the percentages of victims in the quantitative sample who opened up about their experiences to at least one person and the person they disclosed the information to. Most victims did not disclose the sexual victimization. Those who did were most likely to seek informal help (38.5%), and most of them turned to a friend. Few victims sought formal help after sexual victimization, and no one reported to the police.

#### 3.4.2. The Process of Seeking (In)Formal Help upon Sexual Victimization

**Figure 1:**
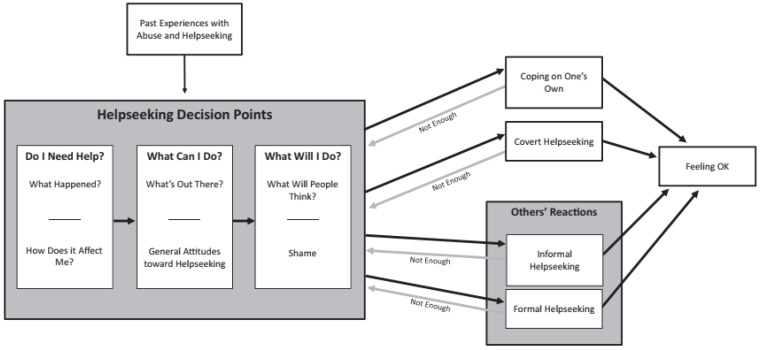
Deciding Where to Turn model. Reprint from Deloveh & Cattaneo (2017) [21].

In what follows, we will present the identified barriers and the decision-making process underlying help seeking strategies of AIP victims of sexual violence organized in line with the Deciding Where to Turn model [21]. This theoretical model (see Figure 1) was developed to explain help-seeking behavior in the general population, but is relevant for the AIP population in Belgium as well, as it may inform us about communalities and differences in the decision-making process of victims from both the AIP and general population. The model explains how victims make decisions about seeking help via three key decision points, namely (1) deciding whether they need help; (2) exploring their options for seeking and receiving help; and (3) weighing the pros and cons of the identified options [21].

The help-seeking decision points identified in this model are influenced by prior experiences with sexual victimization and help-seeking. The decision-making process can result either in coping with the victimization on one’s own, covertly seeking help, or overtly seeking help from either formal or informal sources of help. As long as victims do not feel “ok” they may move back and forth between the key decision points and the coping strategies [21].

*Do I need help?*

All participants in the qualitative study were invited for participation and selected because they indicated they had been exposed to unwanted sexual experiences in the quantitative study. As such, they could all identify the unwanted nature of the incident(s) they were exposed to, although they did not necessarily all label their experiences as sexual violence. Regardless of the label they gave to their experiences, they recognized the impact it had on their lives and that the exposure to (in)direct sexual violence had affected them psychologically, physically, sexually, reproductively, and/or socioeconomically (see Section 3.3). Fatoumata (23 years old) explained that after being in Belgium for a while, she started to feel the need to talk about what had happened as she noticed that her behavior, which was a consequence of the violence she experienced, was often wrongly interpreted by the people around her in the reception center. She said:

“In Africa, I didn’t talk about it, but here [in Belgium] when I arrived, well like I needed someone who could hear me actually, who could understand what…when we are girl, I get angry from around me and they don’t even know what is going on now.”

(Fatoumata, 23 years old)

*What can I do?*

The second key decision point relates to knowing the potential resources that are available and the general attitudes of victims toward seeking help upon victimization. Table 8 presents the barriers for help-seeking after sexual victimization identified in the quantitative study. AIP victims of sexual violence indicated in 4.8% of the cases that they did not know where they could turn to for receiving formal help or that professional help was not accessible because of financial or transportation limitations.

We did not probe in an in-depth manner about the interviewee’s knowledge of resources in this study. However, interviewees did report on their attitudes toward seeking help and their estimation about the accessibility of formal help resources. Most of the interviewed AIP victims recognized that they could benefit from receiving help, but that they would not have access to it because of their legal status, because of a lack of available resources and/or because of negative social consequences associated with seeking help. In addition, waiting lists were mentioned as an important barrier as well. In that regard, Ayman (36 years old) explained how waiting lists in his home country hindered seeking help from a mental health professional: “So, we have for example in the city like Aleppo. I think, there are doctors, so psychologists are there. But you can count them on one, one hand actually. About five, six for a large, for I don’t know how many millions probably of inhabitants. So, before you can get a turn at a psychologist, it takes a very long time.”

The identified barriers influenced their decisions to disclose their experiences with sexual victimization and seek (in)formal help.

*What will I do?*

According to the model of Deloveh and Cattaneo [21], victims of sexual violence will decide to proceed with their search for help and choose the most suited resource. As mentioned earlier, most of the participants in the quantitative study did not seek informal help (61.5%), professional help (96.2%), or help from the police (100%). The reasons for not seeking formal help upon sexual victimization reported in the quantitative study are presented in Table 8.

The most important barriers for seeking help reported by the AIP victims who participated in the in-depth interviews can be grouped under the themes of shame and taboo. The in-depth interviews revealed that in many families and communities, sexual victimization is kept a secret to avoid stigmatization of the victim and their families. Maryam (53 years old), for example, explicitly said: “Which means that there are families that would rather keep it a secret. So that almost never comes out, um. That is a shame.” The stigma and the consequences associated with sexual victimization, result in the subject being made taboo, which hinders direct and indirect victims from disclosing the violence and seeking both (in)formal help. Fatoumata (23 years old) for example tried to hide what had happened because she was not close with her parents and did not know how they would react. She narrated: “Then after this happened, immediately I took off the sheets as I didn’t talk too much with my mother nor with…. I wasn’t able to confide in them because we weren’t close at all, I changed the sheets to hide, I was quick, I took a shower.”

Most AIP victims worried about what others would think about them when learning about their experiences or when they would seek certain types of care (e.g., psychological care). Ali (26 years old) explained that he remained silent because he feared that others would perceive him as less of a man: “He’s [referring to himself] not normal, he’s not man, or not…. I don’t know.”

Elen (48 years old) described how seeking help from a mental health professional was not easy. First of all, because seeking help from a psychologists may (negatively) change the way others perceive a person and secondly, because when AIPs seek help, their underlying motivations are often interpreted through the lenses of the prejudice that AIPs will do anything to get papers. She said:

“I have a lot to say but I can hardly say anything, even to the social workers or to other people, because they always look at you differently. For example, I have a sick daughter now…she really needs a psychologist because she’s just depressed. She’s been through a lot…, but still some people keep saying ‘yeah maybe she’s just doing that because she wants to get papers this way or something’, so that makes you even more…. There are communities that have a bad, a bad impression from the moment a psychologist comes along.”

(Elen, 48)

Many victims also confessed to have remained silent out of fear of not being believed or taken seriously. A lack of trust in others added to the decision of several interviewees to keep the information to themselves. Both Fatoumata and Majohoul testified to how a lack of trust and the fear of negative consequences (i.e., negative emotions and disappointment related to not being believed by her mother in the case of Fatoumata, and being at risk of exploitation in the case of Majohoul) hindered them in seeking help and disclosing the experienced sexual violence.

“I didn’t feel well. I was sick. I didn’t feel well at all, but nobody noticed, but I knew what had happened and then I told my mother. I told her, when she wasn’t there, when Mr. came here there was nobody there and Mr. raped me. My mother she told me ‘Mr. can’t do that,’ how he can’t do that. I told her, ‘Mr. did it to me’ because…she told me ‘No, Mr. didn’t do it’ and I showed her the sheets.”

(Fatoumata, 23 years old)

“So, I never sought help because I was afraid that I…I didn’t trust people anymore. So, I was afraid that if I tell people something, they will always want something from me. So, when I went to the people I was telling my story to, I, I, that’s why I had closed myself off a bit and chosen not to tell anything. So that’s why I was, that’s what I was afraid of.”

(Majohoul, 33 years old)

For some of the interviewees, it felt safer to talk about their sexual violence experiences with people with whom they shared a cultural background or language. Cristiano (25 years old) linked this to the shared cultural background and language. He said: “Of course, I talk about it with Sudanese people who understand me. Because it’s hard to tell external people because they don’t understand your language anyway. So, it’s always the same people who listen to you.”

On the other hand, sharing a certain cultural background was by some also considered as a reason not to discuss their experiences with certain persons. Ali (26 years old) explained it as follows:

“For example, last month I had a visitor, a friend. He’s from Switzerland…. I really, I cried with him, I speak with him a lot. Like, I don’t know, I speak from my heart. I need it. Maybe it was the help I asked…. If I tell this story to… I feel, I don’t know…. Iraqi, Syrian, Palestinian, Moroccan, Irani, Afghan, I speak all their language, so I know their culture also. I will never talk about it with them, or I would never like they know about this.”

(Ali, 26 years old)

Others also indicated that they only disclosed their experiences to our research team or to people they had met in formal or informal contexts in the host country. In the latter case, two reasons were articulated: (1) AIPs expected European citizens to have a different and more “open” viewpoint on sexual victimization than the members of the communities they belonged to, and (2) the interview took place in an objective research context, and they deemed it important to share their victimization experiences to protect potential future victims. Majohoul (33 years old), for example, indicated that he felt the need to discuss his experiences, but that he only felt safe to do so for the first time with the interviewer who conducted the first face-to-face structured interview, which was part of the quantitative study (cf. supra), and that the professional attitude with which the interviewer treated his story, motivated him for further disclosure. Related to this, he said: “No, that was the first time, and when I, I actually gave the first interview, at the end there was this question asked of, ‘Are you willing to talk about that?’, I immediately answered yes, because I really wanted to talk about that”.

Even when one feels generally supported by friends and family, disclosing what happened is sometimes too big of a step to take. For some, like Youssef (31 years old) who said “yes, I tried, of course, to ask friends or acquaintances from Gaza [for help]…. But everyone is busy with their own…. Everyone has their own story so, I was stuck”, it felt as if they would burden others too much. For others, disclosure was also considered as a potential trigger of negative feelings. Ali (26 years old) described how this was for him:

“What I did after, only I, my friend when he sees me like this he say ‘I can do whatever you want, I stay with you’. Of course, we went to this guy [Ali’s friend], to his home, he was, I had his name, and I know him very well, we went to his home, because I feel ashamed to tell my family, to tell my brothers, and also I feel ashamed to tell this friend who is with me. Just I told him… I make fire his [the assailant] car, his home….”

When it comes to barriers for reporting to the police, the reasons are very diverse and very often influenced by previous encounters with the police, not only in host countries such as Belgium, but also in the home country and en route.

Some victims did not seek help from the police because of the image they have about the police. Rahi (42 years old) explained that she does not feel like the police can protect her, or want to protect her, because of her legal status. After a violent attack in Athens, upon reporting, the police had sent her away because she was an AIP: “It was terrible because eh, there the police cannot protect you, because if you go to police, he say ‘you don’t have any right here’.”

Not reporting could also be linked to the migration process. In the case of Majohoul (33 years old), as he was still on the run and didn’t feel safe yet, he wanted to remain under the radar. He judged that going to the police would bring him into more trouble than it would help him. He explained his motivations to not seek help from the police:

“My intention was actually to also remain anonymous and as soon as possible [to leave] Egypt, because my intention was not to stay in Egypt, so the more that you assert yourself, the sooner they [the persons for whom he was fleeing] will get you. So, I, my intention was to flee Egypt too…. Even if I had to go to the police, and okay, I’ll report it. And after that? Hey, that probably wouldn’t work out anyway… so, if they [the persons for whom he was fleeing] had to know about what was going on, they could also do something to me. So, I was actually, I was in a cocoon, I was stuck.”

(Majohoul, 33 years old)

In other cases, not reporting to the police was linked to the victim and the sexual victimization incident itself. Frequently, interviewees mentioned that they did not think the sexual violence was severe enough to seek help or they believed that nothing could be done about it, as with Cristiano (25 years old), who explicitly said: “No, there is actually no one who can help me”. Finally, another returning reason relates to the frequent exposure of AIPs to sexual violence (see Section 3.2).

*Seeking help without disclosure.*

Because the reported sexual victimization disclosure rates are rather low in the quantitative study population (see Table 7), our expectation was that AIP victims would mostly rely on the strategies “coping on one’s own” and “covert help-seeking” described by DeLoveh and Cattaneo [21]. This was confirmed in the in-depth interviews. Most of the interviewees had never told anyone about the sexual victimization they had been exposed to. Those who did tell someone rarely disclosed details. Most of them sought ways to deal with the consequences themselves or by seeking help without revealing the cause of their symptoms. Ali gave two examples of ways in which he sought help in a covert manner:

“And our solution is time, too much time. With God you can be better, but it will be very quiet, very quiet, secret, something like this. They have to…. Maybe I have psychological problems, but I go to my doctor, and I cannot talk to him. I say to him ‘I cannot sleep’, for example, ‘ah, okay, maybe you can try stress tea’. Maybe, because, something, some people, they don’t understand.” He went on to say:

“I just call my friend and I told him something like ‘behead me, they take me’, but I didn’t say to him what happened….”

(Ali, 26 years old)

Attempts to protect themselves from further violence often occurred while remaining silent. Elen (48 years old), for example, noticed that women who wear a headscarf were less harassed in the center she was residing in. She said “at a certain point I also decided, look, I’m going to wear a headscarf from now on, so they’ll leave me alone. So that’s also a form of self-protection”. In this way she inscribed herself into a social group that in her eyes was less at risk of victimization. This way of protecting oneself is in contrast with experiences from other AIP women. Rahi (42 years old), for example, described that when she was harassed on the bus in Belgium, she immediately associated this with her being a target because she was wearing a headscarf. As a Muslim, she had experienced Islamophobic prejudice events in the past. Adopting self-protection strategies thus appears to have variable outcomes, depending on the context and prior victimization experiences.

AIP victims also described the selectivity of their disclosures and how the results of their attempts to seek help led to adjusting their coping strategies. Upon positive experiences, victims tended to share their experiences with more people and/or to give more details of what had happened to them. Negative responses to their disclosures often made them take a step back and return to silence and secrecy. Some AIPs also described mixed feelings about the consequences of seeking (in)formal help as they alternate between positive and negative attributions over time. Fatoumata (23 years old) described how, although therapy had helped her already a great deal, the consequences of talking about her experiences also brought her down at times. At a certain point, she decided that stopping to talk about her experiences would help her to focus more on the future instead of the past. She explained:

“So, when I started to talk, I started to feel good but then I said to myself why aren’t you here [in the present], it’s done anyway, it’s not going to change so you better, well with that, it is over…. I don’t want to look back [in therapy] when I don’t want to think about it anymore. Sometimes I even made progress with my daughters. I don’t want to look back every time…. I don’t want to talk about it. I want to look at my future, what is a possible here, what can I do here, what, me and my daughters how are we going to get out of this. Well, I need to put all this in my head, forget and all that and if it is to talk more and more, no I can’t manage. It’s too much, even in the office I got a little, I got a little lost. I’m tired, but she [the therapist] understood that I’m tired in fact. I could not, no.”

(Fatoumata, 23 years old)

#### 3.4.3. AIPs’ Recommendations to Prevent Sexual Victimization and to Improve Care upon Victimization

During the in-depth interviews, interviewees were asked about what they would recommend to policy makers and care givers to improve prevention strategies and care paths designed to offer help to victims of sexual violence. According to AIPs, the key to both prevention and care is to break the taboo around sexual violence. Almost all respondents suggested that starting early with an age-appropriate sexual education is crucial. Many indicated that they never received comprehensive sexual and relational education and, as such, experienced difficulties in negotiating safe sexual contacts, discussing both healthy experiences of sexuality and sexual violence, among other things. According to the interviewees, proper sexual education should be part of every individual’s upbringing.

Furthermore, AIP victims also suggested that sensing that they would be taken seriously by the police and that they could benefit from police protection could potentially lower the barriers for reporting.

Lastly, they stressed that stigmatization of the victims should end. Safe disclosure should be the norm, not social rejection.

## 4. Discussion

### 4.1. Sexual Violence, Its Impact, and Help-Seeking Behavior upon Sexual Victimization in AIPs

Based on the explorative findings of both the quantitative and qualitative study, we can derive that AIPs residing in Belgium are at high risk to be sexually victimized. Approximately 84% of our sample in the quantitative study reported to have been exposed to some type of sexual victimization in their lifetime, and about 61% reported victimization in the past year. It is important to mention here that, on average, the participating AIPs were residing in Belgium for at least 10.8 months. This means that most participants who reported sexual victimization in the past year might have been victimized in Belgium. However, confirming findings from earlier studies [9,10,13], AIPs residing in Belgium also reported exposure to violence in their country of origin and while on the road. When we look at hands-on sexual violence, almost half of our sample had been victimized in their lifetime, and 1 in 4 reported exposures to rape. The findings from the qualitative study confirm that AIPs are exposed to (in)direct sexual victimization and that they associated it with their legal status and the vulnerability it comes with. Keygnaert et al. [16] found in their study that sexual violence was considered as part of the process of migration, almost as a rite of passage. Based on our in-depth interviews, we learned that our respondents also considered exposure to sexual violence almost as normal because it occurred so often to them and those around them. Although the experiences seem to get normalized as a way of coping with it, sexual victimization’s impact remains undeniable (cf. infra).

In line with previous studies in Belgium [11,12,43], we found that men and women follow the same trends in terms of victimization risks. Moreover, we also found more gender-balanced results related to the gender of the assailants. This is in contrast with findings in the general population where assailants are mainly identified as males [44]. Still, sexual violence remains mainly portrayed in terms of white female girls sexually assaulted by male assailants [34,45,46]. This rape myth reinforces gender binary and heteronormative thinking and ignores the true nature of this societal problem threatening public health [47]. Moreover, when migrant males are predominantly depicted as assailants and their victims as native white females, intersecting gendered dynamics of racism and sexism can be observed in the public discourse [45,47,48]. This discourse leaves aside the particularly vulnerable position of Muslim and minority women [48] and does not correspond with the findings of this study.

In contrast to earlier studies by Keygnaert et al. [11,12], the identified assailants in our study are mostly unknown to the victim. However, authority figures were again identified as a substantive part of the assailants as well [9,16]. This finding is in contrast with what has been found in a national representative study in Belgium, which showed that assailants of sexual violence were mainly known to the victim [44]. This may be linked with our second research question, namely the context in which sexual violence against AIPs takes place. In our study, AIPs described sexual violence exposure predominantly while they were on the move or had arrived in Europe. The bulk of incidents reported concerned (attempts at) sexual exploitation by locals. More research is needed to study potential shifts in the context in which sexual victimization occurs and the involved assailants before and after the European refugee reception crisis in 2015.

Sexual victimization has an important impact on the lives of AIPs. Victims report mainly psychological and socioeconomic consequences. However, mental health did not seem to be impacted by sexual victimization in our quantitative sample. This could be related to the lower power due to the small sample size, but it could also be the result of the general poor mental health statuses observed in this population [49,50]. Many AIPs experienced more than one traumatic event as well as hardship linked to pre- and post-migratory experiences. Poor mental health has been identified in earlier studies as associated with increased sexual victimization incidents (see [15,17,18]).

Furthermore, sexual victimization has also been identified as a reason to leave the country of origin. As a result, victims may lose their support network and as such be more at risk for further violence as both social protection and social support as coping mechanisms are lacking [15,20].

In addition, because AIPs have a lot of worries related to the migratory process impacting their mental health, they are not always immediately able to protect themselves or to identify when someone is taking advantage of them. Adding to existing worries, having to cope with being at risk of sexual victimization can contribute to the mental strain they experience. As such, the vulnerabilities may circularly feed into one another.

This study also shows that disclosure and help-seeking behavior upon sexual victimization seem to follow the decision-making process theoretically described in the “Deciding Where to Turn” model developed by DeLoveh and Cattaneo [21]. Although sexual victimization impacted the lives of the victims in this study significantly, disclosure and help-seeking behavior are very limited in this population. This is likely the result of taboo and stigma related to the violence. In addition, previous negative (in)direct experiences with disclosure, seeking help or reporting to the police form important barriers to seeking help in general. By postponing help-seeking, sexual victimization consequences which are initially temporary in nature, could become chronic or could worsen and may ultimately result in severe complications due to a lack of proper care. Because AIP victims indicated that they did not always know where to turn to seek help and/or experienced barriers for accessing resources related to their legal status, it is essential to invest in initiatives informing them about their right to health care and supporting them in navigating the health care system. Moreover, it is essential to create safe contexts for disclosure. The prior negative experiences of AIP victims not only hinders the reporting of sexual violence, but also potentially decreases their trust in the potential outcomes and benefits of judicial processes. Considering that AIPs may struggle with trusting professionals and police because of earlier experiences, taking time to gain trust seems essential to lower those barriers and to improve care upon sexual victimization. To this end, positively changing the image of police and allowing safe reporting by using firewall measures [51] could increase reporting. In addition, diversity-sensitive communication is currently not included in the basic training curriculum of any type of professional working with victims [52,53,54,55,56,57]. This transferable skill should be integrated in higher education programs and providing continued education to further develop this skill in professionals is urgently needed as well [52,53,54,55,56,57]. Finally, because AIPS in this study testified that they had rarely received comprehensive relational and sexual education, informing professionals about existing sensitization programs (e.g., Senperforto Frame of Reference [58]) and training them to implement these programs when AIPs arrive in Belgium, is essential to help reduce the high numbers of reported sexual victimization in AIPs and to respond adequately when they come into contact with a (potential) victim.

### 4.2. Strengths, Limitations, and Suggestions for Future Research

This explorative mixed-method study is strengthened with the applied triangulation of data. First, AIP participants in the qualitative study cross-culturally defined sexual violence in line with the central sexual violence definition in our study and correspondents with the behaviorally specific questions that were used in the quantitative survey. This match validates the use of this type of questioning in AIP populations. Future population studies on sexual violence could thus apply the same type of questions in the general population and in AIP populations, increasing the possibilities for comparison between the populations. Secondly, the highly reported occurrence of sexual victimization in the quantitative study was complemented by contextualization through the qualitative data. Our respondents testified being exposed to sexual victimization often, either directly or indirectly in their home countries, when on the move, or upon arrival in Europe, and in this case Belgium specifically. Thirdly, combining the two datasets allowed us to assess how often victims seek help upon victimization and the barriers for seeking help, while understanding how these numbers can be understood in a more in-depth manner.

Like any research, this study came with limitations. Although it was initially designed to overcome the identified challenges in prior prevalence and population research regarding sexual violence in AIPs in Europe [6,28], COVID-19-related sanitary measures forced us to stop the research, which led to our sample being too limited to extrapolate our findings to all AIPs residing in Belgium. However, the high percentages of sexual victimization found in this study highlight the importance of continuing sexual violence research in this vulnerable population. Another limitation to this study relates to the applied mental health scales. Further research is needed to develop and validate mental health scales that provide reliable scores for participants with different cultural backgrounds in different languages. Furthermore, the grooming and sexual exploitation of AIPs of all genders by both male and female Belgian citizens in those places where AIPs reside while waiting to apply for international protection should be further researched to inform policy makers about the situation and formulate recommendations to protect people in vulnerable situations.

## 5. Conclusions 

AIPs of all genders residing in Belgium seem very vulnerable to sexual victimization both before and during the different stages of their migratory process, including upon arrival in Europe and Belgium. Their exposure to sexual victimization rises to almost 84%, and in about 61% of the AIPs, this happened in the last 12 months when they were most likely already in Europe and Belgium in particular.

Although sexual violence impacts AIPs’ lives and health significantly, disclosure and overt help-seeking upon sexual victimization from informal networks, professionals, and/or police were rare. Identified barriers to seeking help are mainly linked to stigma and shame, but also to previous negative help-seeking experiences. Not seeking help in the latter cases is primarily a strategy to avoid further victimization and negative consequences.

## 6. Recommendations

When developing prevention strategies targeting sexual violence in AIPs and care paths aiming to offer adequate care to AIP victims of sexual violence, attention should go to breaking the taboo around sexual violence. Furthermore, investing in age-appropriate relational and sexual education to people of all ages and cultural backgrounds including sex-positive messages and focusing on developing skills to both setting and respecting boundaries, is urgently needed. Measures to lower barriers to seeking (in)formal help should be prioritized, including creating safe disclosure contexts and tackling the stigma victims of sexual violence are facing. Lastly, more research is needed to further scientifically substantiate the findings of this study and related recommendations.

## Figures and Tables

**Table 1:** Sociodemographic characteristics of the study population (*n* = 62).

Variable		M(SD), Range	*n*	%	% SV	M(SD), Range SV
Sex at birth	Female	-	15	24.2	80.0	-
	Male	-	47	75.8	85.1	-
Age in years		32.5 (10.2), 16–61	-			32.4 (9.5), 16–61
Months in Belgium		10.8 (11.2), 0–60	-			9.9 (9.5), 0–36
Education level	Primary or none	-	14	22.6	78.6	-
	Secondary	-	25	40.3	80.0	-
	Higher	-	23	37.1	91.3	-
Occupational status	Active ^a^	-	6	9.7	100.0	-
	Student	-	20	32.3	85.0	-
	Inactive or other ^b^	-	36	58.1	80.6	-
Sexual orientation	Heterosexual	-	55	88.7	83.6	-
	Non-heterosexual ^c^	-	6	9.7	100.0	-

^a^ Participants who are employed/independent, perform voluntary work or work as a contributing family member. ^b^ Participants who are homemaker, on the job market, not able to work because of ill health, financial self-sufficiency, or any other type of alternative choice of living or other. ^c^ Participants who labelled themselves as lesbian, gay, bisexual, omni-/pansexual, asexual, or other.

**Table 2:** Lifetime sexual victimization and sexual victimization in the past 12 months.

	Lifetime		Past 12-Months	
**Item**	*N*	%	*n*	%
**Any sexual victimization**	52	83.9	38	61.3
**Any hands-off sexual victimization**	49	79.0	36	58.1
Sexual staring	30	48.4	17	27.4
Sexual innuendo	20	32.3	14	22.6
Showing sexual images	27	43.5	22	35.5
Sexual calls or texts	25	40.3	16	25.8
Voyeurism	5	8.1	2	3.2
Distributing sexual images	1	1.6	0	0.0
Exhibitionism	17	27.4	9	14.5
Forcing to show intimate body parts	8	12.9	3	4.8
**Any hands-on sexual victimization**	29	46.8	13	21.0
**Any sexual abuse**	27	43.5	12	19.4
Kissing	21	33.9	8	12.9
Touching in care	16	25.8	7	11.3
Fondling/rubbing	15	24.2	8	12.9
Forced undressing	8	12.9	8	12.9
**Any rape**	15	24.2	5	8.1
Oral penetration	3	4.8	1	1.6
Attempt of oral penetration	10	16.1	2	3.2
Vaginal or anal penetration	7	11.3	1	1.6
Attempt of vaginal or anal penetration	7	11.3	1	1.6
Forcing to penetrate	4	6.5	1	1.6

**Table 3:** Nature of lifetime sexual victimization and victimization in the past 12 months per sex at birth.

	Females (*n* = 15)		Males (*n* = 47)		Fisher’s Exact Test	
Type Sexual Victimization	Lifetime *n* (%)	Past 12 Months *n* (%)	Lifetime *n* (%)	Past 12 Months *n* (%)	Lifetime SV	Past 12 Months SV
Any sexual victimization	12 (80.0)	8 (53.3)	40 (85.1)	30 (63.8)	0.693	0.548
Any hands-off sexual victimization	12 (80.0)	8 (53.3)	37 (78.7)	28 (59.6)	1.000	0.767
Any hands-on sexual victimization	8 (53.3)	3 (20.0)	21 (44.7)	10 (21.3)	0.767	1.000
Sexual abuse	7 (46.7)	3 (20.0)	20 (42.6)	9 (19.1)	1.000	1.000
Rape	6 (40.0)	2 (13.3)	9 (19.1)	3 (6.4)	0.163	0.587

Fisher’s exact test (instead of Chi-square test): *p*-value.

**Table 4:** Identified gender of the assailant of sexual violence.

Gender	Lifetime Sexual Victimization (*n* = 46) (%)	Past 12 Months Sexual Victimization (*n* = 38) (%)
Man	41.3	36.8
Woman	41.3	39.5
Unknown	17.4	23.7

**Table 5:** Relationship to the assailant of sexual violence.

	Lifetime Sexual Victimization (*n* = 52) (%)	Past 12 Months Sexual Victimization (*n* = 38) (%)
(Ex)Partner	9.6	7.9
Family member	5.8	7.9
Friend	5.8	34.2
Date	26.9	13.2
Authority figure	7.7	10.5
Colleague/Classmate	19.2	23.7
Acquaintance	17.3	18.4
Unknown	59.6	63.2

Note: Participants could indicate multiple options, therefore the total is >100%.

**Table 6:** Descriptive statistics of mental health status and sexual victimization (*n* = 62).

Item	Scale	Outcome	N	%	% SV	χ^2^/t; df; *p*-Value
Depression	PHQ-9	No	14	22.6	85.7	0.775; 60; 0.441 *
	(α = 0.829)	Mild	20	32.3	80.0	
		Moderate	14	22.6	100.0	
		Moderately severe	8	12.9	75.0	
		Severe	6	9.7	66.7	
Anxiety	GAD-7	No	15	24.2	86.7	1.684; 60; 0.097 *
	(α = 0.857)	Mild	21	33.9	95.2	
		Moderate	24	22.6	71.4	
		Severe	12	19.4	75.0	
PTSD	PC-PTSD-5	Yes	35	56.5	85.7	0.735°
Hazardous alcohol use	AUDIT-C	Yes	7	11.3	100.0	0.586°
Sedative use	NA	Lifetime	29	46.5	86.2	0.738°
		Past 12 months	21	33.9	85.7	1.000°
Cannabis use	NA	Lifetime	13	21.0	92.3	0.673°
		Past 12 months	13	21.0	92.3	0.673°
Illegal drug use	NA	Lifetime	1	1.6	0.0	0.161°
		Past 12 months	0	0.0	0.0	/
Suicide attempt	NA	Lifetime	9	14.5	88.9	1.000°
		Past 12 months	9	14.5	88.9	1.000°
Self-harm	NA	Lifetime	10	16.1	70.0	0.343°
		Past 12 months	7	11.3	57.1	0.076°

α, Cronbach’s alpha; SV, sexual victimization; df, degrees of freedom; PTSD, posttraumatic stress disorder. Patient Health Questionnaire-9 (PHQ-9): mild (5–9), moderate (10–14), moderately severe (15–19), severe (≥20); General Anxiety Disorder-7 (GAD-7): mild (5–9), moderate (10–14), severe (≥15); Primary Care PTSD Screen for DSM-5 (PC-PTSD-5): Yes (≥3); Alcohol Use Disorder Identification Test Short version (AUDIT-C): Yes (≥4 for females, ≥5 for males); % SV: proportion of sexual victimization within the different categories of depression, anxiety, resilience, PTSD, hazardous alcohol use, sedative use, cannabis use, illegal drug use, suicide and self-harm. * Independent sample *t*-test with equal variances assumed (instead of Chi-square-test): t; df; *p*-value.° Fisher’s exact test (instead of Chi-square test): *p*-value.

**Table 7:** Disclosure and help-seeking after sexual victimization.

Variable		*n* (%)		*n* (%) ^a^
Disclosure ^b^	Yes	20 (38.5)	Partner	2 (10.0)
(*n* = 52)			Parent	2 (10.0)
			Other family member	5 (25.0%)
			Friend	14 (70.0%)
			Acquaintance	2 (10.0%)
Professional help	Yes	2 (3.8%)	Medical specialist (no psychiatrist)	1 (50.0%)
(*n* = 52)			Mental health care worker (incl. psychiatrist)	1 (50.0%)
Police	Yes	0 (0%)		
(*n* = 46)				

Note. These results concern the sexual violence incident with the most impact on the victim. ^a^ Participants could indicate multiple options; therefore, the total is >100%. ^b^ Disclosure, disclosure prior to the interview.

**Table 8:** Barriers for help-seeking after sexual victimization.

Item	*n*	%
**Barriers to Contact Professional Help (*n* = 42)**		
Reasons linked to the victim I didn’t need help. I thought nothing could be done. I felt embarrassed about what happened. I would not be believed or taken seriously. I didn’t trust anyone.	25	59.5
Reasons linked to others I was afraid of further violence. I didn’t want the person who did this to me to get in trouble. I didn’t want to bring a bad name to the family or group I belong to.	9	21.4
Reasons linked to accessibility I didn’t know where to go. I wasn’t able to go due to financial or transportation limitations.	2	4.8
Other reasons	6	14.3
**Barriers to contact police (*n* = 46)**		
Reasons linked to the victim It was not severe enough. I felt embarrassed about what happened. I felt partly responsible for what had happened. I did not know what would happen after I told the police.	21	45.7
Reasons linked to the assailant The one who did this to me was someone I know.	5	10.9
Reasons linked to accessibility It was difficult to get to the police or to contact them.	2	4.3
Reasons linked to the police The police would not believe me or take me seriously. The police would not do anything. The who did this to me would not get caught or get punished. I have had previous negative experiences with the police. I felt endangered at the police.	9	19.6
Other reasons	9	19.6

Note. These results concern the sexual violence incident with the most impact on the victim.

## Data Availability

The dataset supporting the conclusions of this article is available from the authors upon reasonable request.

## Supplementary Materials

The following supporting information can be downloaded at: https://www.mdpi.com/article/10.3390/ijerph191912889/s1. Supplementary S1. Detailed outcome measurements sexual victimization.
